# An approach to estimating prognosis using fractional polynomials in metastatic renal carcinoma

**DOI:** 10.1038/sj.bjc.6603192

**Published:** 2006-05-30

**Authors:** P Royston, M Reitz, J Atzpodien

**Affiliations:** 1 MRC Clinical Trials Unit, Cancer Division, Medical Research Council (MRC), 222 Euston Road, London NW1 2DA, UK; 2 Europäisches Institut für Tumor Immunologie und Prävention (EUTIP), Bonn, Germany; 3 Fachklinik Hornheide an der Universität Münster, Dorbaumstr. 300, 48157 Münster, Germany; 4 Medizinische Hochschule Hannover, Carl-Neuberg-Str. 1, 30625 Hannover, Germany

**Keywords:** renal cell carcinoma, immunotherapy, fractional polynomials, prognosis, risk

## Abstract

We present a prognostic model for metastatic renal cell carcinoma based on fractional polynomials. We retrospectively analysed 425 metastatic renal cell carcinoma patients treated with subcutaneous recombinant cytokine-based home therapies in consecutive trials. In our approach, we categorised a continuous prognostic index produced by the multivariable fractional polynomial (MFP) algorithm, using a strategy in which continuous predictors are kept continuous. The MFP algorithm selected five prognostic factors as significant at the 5% level in a multivariable model: lymph node metastases, liver metastases, bone metastases, age, C-reactive protein and neutrophils. The MFP model allowed us to divide patients into four risk groups achieving median overall survivals of 38 months (low risk), 23 months (low intermediate risk), 15 months (high intermediate risk) and 5.6 months (high risk). Our approach, based on categorising a continuous prognostic index produced by the MFP algorithm, allowed more flexibility in the determination of risk groups than traditional approaches.

For renal cell carcinoma, certain prognostic staging factors, notably, performance status, disease-free interval, erythrocyte sedimentation rate (ESR), lactate dehydrogenase (LDH), neutrophils, haemoglobin, extrapulmonary and bone metastases, and number of metastatic sites were identified as prognostic factors for survival (Elson *et al*, 1988; Palmer *et al*, 1992; Lopez-Hänninen *et al*, 1996; Gelb, 1997; Culine *et al*, 1998; Hoffmann *et al*, 1999; Motzer *et al*, 1999, 2002; Atzpodien *et al*, 2003).

However, the importance of each predictor varies from study to study and is, therefore, controversial. Besides heterogeneity in patient populations and treatments between different studies, a substantial reason for the observed variation might be attributable to an inadequate use of statistical methods (Simon and Altman, 1994).

Most researchers who develop and publish prognostic models in cancer seem to assume that to introduce continuous predictors, such as age and haemoglobin, into a multivariable statistical model, it is necessary first to ‘categorise’ the predictors into two groups. However, the choice of an appropriate cutpoint is not usually obvious *a priori*. To avert the worry that an arbitrary choice may be sub-optimal, there have been strategies searching for the ‘optimal’ cutpoint for each continuous predictor, thus, yielding the smallest *P*-value when testing the effect of the categorised predictor in a univariate Cox model or log-rank analysis. Once such a set of cutpoints has been found, the final multivariable model is often determined by applying a standard algorithm, such as stepwise selection of variables, to the candidate predictors. Sometimes, only those individually significant at the 5% level are considered as candidates for inclusion in the multivariable model.

The disadvantages of such a modelling strategy have been rehearsed quite often in the statistical literature. Here, we will illustrate an alternative strategy in which continuous predictors are kept continuous, and in which, furthermore, nonlinear relationships (if present) are detected and modelled appropriately. As it is clearly sensible to derive prognostic groups for clinical purposes and for displaying results, the final step of our approach is to categorise the prognostic index from the final model and to compare it with a traditional approach based on categorised covariates (Atzpodien *et al*, 2003).

## PATIENTS AND METHODS

### Patients

A total of 425 patients with progressive metastatic renal cell carcinoma were entered into consecutive clinical trials between November 1988 and February 1998 to receive either (A) IFN-*α*2a, IL-2 (*n*=102 pts), (B) IFN-*α*2a, IL-2 and 5-FU (*n*=235 pts) or (C) IFN-*α*2a, IL-2 and 5-FU combined with 13cRA (*n*=88 pts) (Atzpodien *et al*, 2003). Median follow-up of these patients was 20+ months (range 0−157+ months). Patient pretreatment included radical tumour nephrectomy (*n*=412), chemotherapy (*n*=5), immunotherapy (*n*=47), chemoimmunotherapy (*n*=8), and hormone therapy (*n*=32).

Criteria for entry into the study were histologically confirmed metastatic renal cell carcinoma, an expected survival duration of more than 3 months, Karnofsky performance status >80%, age between 18 and 80 years, white blood cell count >3500 *μ*l^−1^, platelet count >100 000 *μ*l^−1^, hematocrit >30%, serum bilirubin and creatinin <1.25 of the upper normal limit. Exclusion criteria included evidence of congestive heart failure, severe coronary artery disease, cardiac arrhythmias, symptomatic central nervous system (CNS) disease or seizure disorders, human immunodeficiency virus infections or positivity for hepatitis B surface antigen or chronic hepatitis, or concomitant corticosteroid therapy. In all patients treated, no chemotherapy, immunomodulatory treatment or steroid therapy had been performed during the previous 4 weeks. Pregnant and lactating woman were excluded.

The clinical studies were approved by the institutional review board of the Medizinische Hochschule Hannover; written informed consent was obtained from all patients before entry into the study.

### Treatment design

Treatment regimens were designed to be administered in the outpatient setting. All patients received outpatient subcutaneous (s.c.) IFN-*α*2a and s.c. IL-2. Treatment A consisted of s.c. rIFN-*α*2a (Roferon®, Hoffmann-La Roche; Grenzach-Wyhlen, Germany) (5 × 10^6^ IU m^−2^, day 1, weeks 1+4; days 1, 3, 5, weeks 2, 3, 5, 6 and s.c. rIL-2 (Proleukin®, Chiron, Emeryville)) (10 × 10^6^ IU m^−2^, twice daily days 3–5, weeks 1+4; 5 × 10^6^ IU m^−2^, days 1, 3, 5, weeks 2, 3, 5, 6), only; weeks 7 and 8 were therapy-free. Treatment B consisted of IFN-*α*2a (5 × 10^6^ IU m^−2^, day 1, weeks 1+4; days 1, 3, 5, weeks 2+3; 10 × 10^6^ IU m^−2^, days 1, 3, 5, weeks 5–8), IL-2 s.c. rIL-2 (10 × 10^6^ IU m^−2^, twice daily days 3–5, weeks 1+4; 5 × 10^6^ IU m^−2^, days 1, 3, 5, weeks 2+3) and intravenous (i.v.) 5-FU (1000 mg m^−2^, day 1, weeks 5–8 (Atzpodien *et al*, 2004)). Treatment C consisted of treatment arm B combined with po 13cRA (20 mg 2 × daily over 8 weeks). Eight-week treatment cycles were repeated for up to three courses unless progression of disease occurred. Concomitant medication was given as needed to control adverse effects of chemoimmunotherapy.

### Fractional polynomials

Fractional polynomials were introduced by Royston and Altman (1994) as an extension of the familiar and well-established polynomial method of modelling with continuous predictors. The aim was to increase the range of functions that could be represented, while maintaining simplicity and mathematical tractability. We will denote by *x* a continuous prognostic factor. By transforming *x*, the first-order polynomial (i.e. linear function)

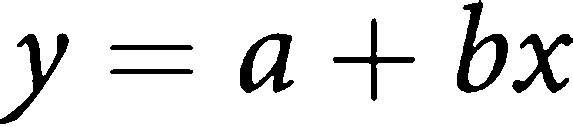
 
is extended to the first-order fractional polynomial or FP1 function

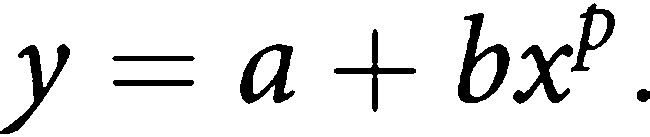
 
For technical reasons the power *p* is restricted to the special set −2, −1, −1/2, 0, 1/2, 1, 2, 3. Here ‘*x*^*0*^’ denotes the natural log function, log(*x*). The second order or quadratic polynomial

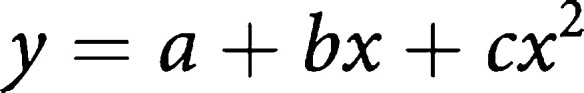
 
is extended to the second order fractional polynomial or FP2 function

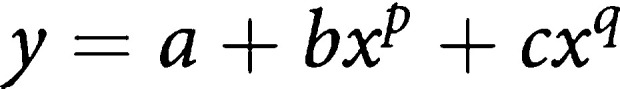
 
or

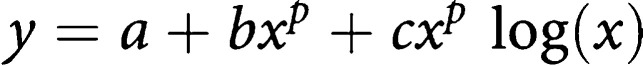
 
the second form being known as a ‘repeated powers’ model. Royston and Altman (1994) demonstrated that by varying the powers (*p*, *q*) and the coefficients (*b*, *c*), a remarkable range of curve shapes could be created from these simple families of mathematical functions. This imparts great flexibility for modelling nonlinear relationships in real data. Note that linear and quadratic polynomials are special cases of the more general FP1 and FP2 fractional polynomials.

Technical details of how a fractional polynomial model for one predictor *x* is determined (that is, how the powers are estimated) and how significance testing is done will not be given here; interested readers are referred to Royston and Altman (1994). An introduction to fractional polynomials in the context of estimating the prognosis of breast cancer patients is given by Sauerbrei *et al* (1999).

### Multivariable modelling with fractional polynomials

Sauerbrei and Royston (1999) developed the MFP (multivariable fractional polynomial) approach to building models from several predictors of which at least one is continuous. They exemplified the method in prognostic and diagnostic modelling in breast cancer.

Thus, we first specify a nominal *P*-value for testing for inclusion of variables and for determining the complexity of the functional form for all continuous predictors. The conventional 0.05 level was used. The algorithm then works in an iterative fashion, sorting out which are the significant predictors and how much simplification of the functional forms can be made at the given significance level. The simplest function for a given continuous predictor is a straight line, and this is chosen by default if there is no convincing evidence of nonlinearity.

The final model in survival analysis can then be used to produce a prognostic index or risk score, which is a weighted combination of the predictors with weights (regression coefficients) taken from the Cox model. The prognostic index value for a given individual summarises the relative hazard of that person with respect to a hypothetical patient with predictor values all equal to zero. If a fractional polynomial is required for any predictor, that function was used when calculating the prognostic index.

An example of the form of a prognostic index for three variables *x*_1_, *x*_2_, *x*_3_ is as follows:

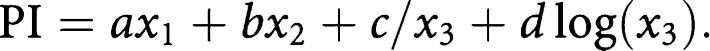
 
Here, the continuous predictor *x*_3_ has been transformed with an FP2 function with powers −1, 0, whereas the other two predictors are included as linear functions. The constants *a*, *b*, *c* and *d* are determined by fitting the Cox model to the data in the usual way. Software to run the MFP algorithm is available in the packages Stata, SAS and R (see Sauerbrei *et al*, 2006 for details).

Survival was measured from start of therapy to date of death or to the last date known to be alive. Survival curves were estimated by the Kaplan–Meier method.

## RESULTS

We constructed a prognostic model based on a study of 425 metastatic renal cell carcinoma patients using fractional polynomials.

Six binary (sex, lung, lymph node, liver, bone, brain/CNS metastases) and eight continuous (age, time from diagnosis to metastatic disease, number of metastatic sites, ESR, C-reactive protein (CRP), haemoglobin, neutrophils, LDH) predictors were included in *univariate* FP analysis.

The MFP algorithm selected five prognostic factors as significant at the 5% level in a multivariable model: lymph node metastases, liver metastases, bone metastases, age, CRP and neutrophils (Table 1). C-reactive protein was subject to an FP1 transformation with power −2.

**Figure 1:**
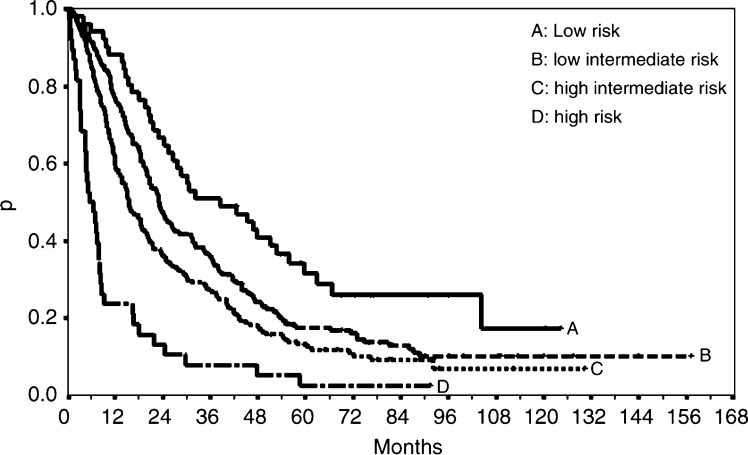
Kaplan–Meier curves for four risk groups based on prognostic index from MFP model. Groups contain 10, 40, 40 and 10% of events, respectively.

Figure 1 shows the survival curves when the patients were divided into four risk groups. These were chosen by applying cutpoints to the prognostic index at the 10th, 50th and 90th centiles, for the subset of patients who experienced an event (i.e. who died). The formula for the prognostic index from the final Cox model was as follows:

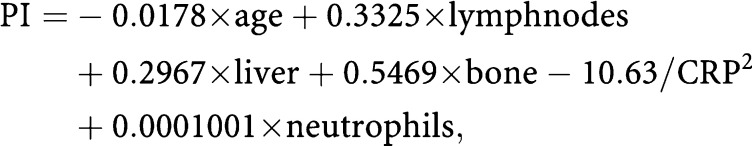
 
where ‘lymphnodes’ takes the value 1 for patients with lymph node metastases and 0 for patients without lymph node metastases, similarly ‘liver’ for liver metastases and ‘bone’ for bone metastases. The cutpoints to divide the patients into the four prognostic groups are −0.986, −0.476 and 0.147. For example, consider a patient aged 50 years, with lymph node metastases but no liver and bone metastasis, CRP 23 mg l^−1^ and neutrophils 3000 (cells *μ*l^−1^). The *PI* is −0.0178 × 50+0.3325 × 1+0.2967 × 0+0.5469 × 0–10.63/23^2^+0.0001001 × 3000=−0.2772 placing them in risk group 3 (between the 50th and 90th centiles of risk).

Median overall survival for low (*n*=51), low intermediate (*n*=172), high intermediate (*n*=164), and high-risk (*n*=38) patients was 38 months (95% CI: 24, 53), 23 months (95% CI: 20, 27), 15 months (95% CI: 13, 20), and 5.6 months (95% CI: 4.5, 7.9), respectively (Table 2).

## DISCUSSION

During recent years, many prognostic factors (clinicopathological, biological, molecular) have been investigated to construct prognostic models that predict the clinical course of renal cell carcinoma patients (Elson *et al*, 1988; Motzer *et al*, 2002; Atzpodien *et al*, 2003). However, the importance of individual factors is controversial. One important reason for this concerns the inadequate use of statistical methods for continuous predictors, such as age or haemoglobin, finally leading to difficulties in comparing multivariable models with different categorisations of the factors (Simon and Altman, 1994).

Although our retrospective data set did not fully comply with the recent publication of REMARK guidelines for prognostic studies (McShane *et al*, 2005), here, we illustrated an alternative and very effective method for selecting a prognostic model in which continuous predictors are kept continuous, and in which, nonlinear relationships are detected and modelled appropriately by using fractional polynomials.

Based on our data set of 425 metastatic renal cell carcinoma patients, the MFP algorithm selected six prognostic factors as significant at the 5% level in a multivariable model: lymph node metastases, liver metastases, bone metastases, age, CRP and neutrophils. The model of Atzpodien *et al* (2003) based on the same data set but using the log-rank test for categorical variables and a score based on Cox proportional hazards model, had in common the predictors CRP, neutrophils and bone metastases; however, they excluded age, lymph node metastases and liver metastases and included instead LDH, time from diagnosis to metastatic disease, and number of metastatic sites. The last three predictors, according to the MFP algorithm presented here, had *P*-values of 0.1, 0.5 and 0.09, respectively, after including the six significant factors.

The beauty of developing a continuous prognostic index, as presented here, is that it provides enormous flexibility in creating risk groups. The sample of patients may be divided into any number of equal or unequal groups. The only major caveat is that creating a large number of groups is unlikely to reliably divide the population into such fine risk strata. Usually no more than three or at most four groups should be produced. Thus, we divided the patients into four risk groups chosen by applying cutpoints to the prognostic index at the 10th, 50th and 90th centiles, for the subset of patients who experienced an event (i.e. who died). Evening out the number of events in this way tends to give more reliable risk estimates, as the number of events is the effective sample size in a survival analysis. Low (*n*=51), low intermediate (*n*=172), high intermediate (*n*=164), and high-risk (*n*=38) patients exhibited a median overall survival of 38 months, 23 months, 15 months and 5.6 months, respectively. In comparison, the median survival times for the three risk groups derived from the same data set by Atzpodien *et al* (2003) were 32 months (low risk), 18 months (intermediate risk) and 8 months (high risk).

A measure of prognostic discrimination that is sometimes advocated is the c-index (Harrell 2001, p. 493), a generalisation for survival data of the area under the receiver–operator characteristic (ROC) curve. The three-group classification of Atzpodien *et al* (2003) had a c-index of 0.628, compared with 0.632 for the model derived by the MFP algorithm. The discrimination is therefore much the same between the two models. According to the measure of explained variation proposed by Royston and Sauerbrei (2004), the *R*^2^ values for these two models are both 0.11.

In conclusion, the method based on a continuous prognostic index was apparently able to refine the classification of survival times in the present renal cell carcinoma patients. Although, in our data set the prognostic capacity of the two models was similar, the approach based on categorising a continuous prognostic index produced by the MFP algorithm allowed more flexibility in the determination of risk groups.

It should be noted, though, that the present data set was of good performance status patients, fit enough for immunotherapy. The results are not necessarily generalisable to the whole population of metastatic renal cell cancer patients. The acid test of any model is its ability to predict well in independent data. As a data set with similar prognostic factors measured was not available to us, we could not yet evaluate the generalisability of the two models. Validation of our proposed model will require testing in a prospectively designed study of a wider patient population.

## Figures and Tables

**Table 1:** The MFP algorithm applied to the renal cancer data set

**Predictor**	**In/out of model**	***P*-value^a^**	**FP transformation**
*Binary*			
Sex	Out	0.8	
Lung metastases	Out	0.9	
Lymph node metastases	In	0.005	N/A
Liver metastases	In	0.05	N/A
Bone metastases	In	<0.001	N/A
Brain/CNS metastases	Out	0.2	

*Continuous*			
Age	In	0.02	Linear
Time from diagnosis to metastatic disease	Out	0.4	
Number of metastatic sites	Out	0.3	
ESR	Out	0.3	
CRP	In	0.01	FP1 (−2)
Haemoglobin	Out	0.6	
Neutrophils	In	0.001	Linear
LDH	Out	0.2	

a At final cycle of the MFP algorithm.

CNS=central nervous system, CRP=C-reactive protein, ESR=erythrocyte sedimentation rate, LDH=lactate dehydrogenase, MFP=multivariable fractional polynomial.

**Table 2:** Median survival by risk group from the MFP model

**Risk Group**	**Number of patients/deaths**	**Median survival (months)**	**95% confidence interval**
Low	51/37	38	24, 53
Low intermediate	172/149	23	20, 27
High intermediate	164/148	15	13, 20
High	38/37	5.6	4.5, 7.9
